# Role of sequence of feeding on the properties of polyurethane nanocomposite containing halloysite nanotubes

**DOI:** 10.1080/15685551.2019.1687083

**Published:** 2019-11-22

**Authors:** Hadi Oliaie, Vahid Haddadi-Asl, Mohammad Masoud Mirhosseini, Iman Sahebi Jouibari, Sara Mohebi, Arash Shams

**Affiliations:** Department of Polymer Engineering and Color Technology, Amirkabir University of Technology (Tehran Polytechnic), Tehran, Iran

**Keywords:** Thermoplastic polyurethane, in-situ polymerization, halloysite nanotube, phase separation

## Abstract

Polyurethane/Halloysite Nantubes nanocomposites containing 1 wt.% nanoparticles were prepared using in situ polymerization method with different mixing sequences. Various experiments have been performed in order to evaluate the effect of nanoparticle dispersion and the different orders of mixing of the samples on the mechanical properties and morphology of nanocomposites. The results obtained from the ATR-FTIR test demonstrated that the presence of nanoparticles led to an increase in phase separation, and the sample with the best nanoparticle dispersion has shown more phase separation than the other samples. Furthermore, the results of the Differential scanning calorimetry (DSC) also confirmed more phase separation and the crystallinity of the samples in the presence of nanoparticles. Scanning electron microscope (SEM) images were utilized in order to investigate the dispersion of nanoparticles in polyurethane matrix and to examine surface fracture of the samples. Moreover, differential mechanical thermal analysis (DMTA) revealed that the presence of nanoparticles has altered the glass transition temperature of polymers, and there are physical and chemical interaction and hydrogen bonding between nanoparticles and hard and soft polyurethane segments. In addition, in the presence of nanoparticles the damping of the samples was reduced compared to the neat sample. Change in behavior from liquid like to solid like in the range of low angular frequencies was observed which is in agreement with the formation of a network structure that can be broken even at low shear rates. In the second step, kinetics of the phase separation process of thermoplastic polyurethane and nanocomposites was studied by rheological experiments. The results showed that the kinetics of phase separation process of thermoplastic polyurethane is similar to that of the crystallization process. Phase separation kinetics of neat samples and nanocomposite have been studied. The presence of nanoparticles by nucleation mechanism increased the rate of the phase separation.

## 1. Introduction

Polyurethanes commonly referred to as urethane, are a relatively new collection of highly resinous polymers that have grown rapidly over the past thirty years to produce sponges, synthetic fibers, coatings, adhesives, paints, and elastomers. Thermoplastic Polyurethanes are copolymers with a linear structure consisting of soft and hard blocks. In fact, in this copolymer, a piece of each segment of the chain of soft and fairly long segments is linked by hard segments through a covalent bond. Due to the difference in polarity between soft segments and hard segments, the thermodynamic incompatibility of in these segments results in phase separation. soft segment molecular weight [1,2], diisocyanate symmetry [3,4], ability Hydrogen bonding ability [5] and the methods for synthesizing and processing polyurethane [6–9] are some factors that affected phase separation [10]. Peddrzoli et al. [11] found that carbon nanotubes are effective in the hard and soft phase of the polyurethane morphology, and their combination with cellulosic nanocrystals can have the greatest effect.

The addition of CNT to the polyurethane results in the formation of small, larger cells. This is due to the presence of uncrystallizable areas that prevent the growth of crystals in the cortex and stop their growth [12]. yung et al. [13] investigated the effect of mixing order on the preparation of polyurethane nanocomposites and showed that modified CNTs have good compatibility with polymer matrix. In addition, nanoparticles tend to have a dispersed distribution over random distribution in a polymer matrix [14]. Yu et al. has investigated the effect of the method of synthesis of polyurethane nanocomposites on nanoparticle dispersion and the mechanical properties of nanocomposites, and concluded that in-situ polymerization is a better distribution of nanoparticles [15]. Chou et al. [16] used the insitu polymerization method for the synthesis of TPU/Clay nanocomposites and found that the order-disorder temparature in the presence of silica nanoparticles increased. In the research of Bera et a [17]., TPU nanocomposites have been prepared by in situ polymerization and GO and RGO nanoparticles have been grafted onto polyurethane chains. In the nanocomposites, a decrease in Tg is observed, but in higher percentages Tg is increased [3]. Also, more interactions in the hard phase due to their more functional groups, phase separation occurs in these nanocomposites. Chen et al. Also reported this process as a polyurethane phase separation [18].

The chemical formula is Halloysite (Al2Si2O5 (OH) 4.nH2O), which is the only difference with the kaolin formula that there is more structural water than it is [19]. The high aspect ratio of the nanoparticle facilitates the load transfer from the matrix to the nanotubes, thereby helping to strengthen the polymer matrix [20]. Adding a small amount of Halloysite nanotubes (HNTs) by accelerating the crystallization speed accelerates the processing time and significantly increases the thermal resistance of the nanosized composite [21]. Due to the high dispersion of Halloysite nanoparticles, these nanoparticles can be dispersed in thermoplastic matrices, especially polar polymers, such as polyamides, by melting in a uniformly uniform manner [22]. Liu et al. [23] experiments on copolymer carboxylated styrene/butadiene and Halloysite copolymer composites were performed. It was found that by increasing the weight percent of Halloysite in nanocomposites, a significant increase in the mechanical properties of nanocomposites would be expected. Heng et al. [24] showed that tensile strength, and impact strength increase with increasing weight percentage of HNT nanoparticles. Other studies conducted by Ye and Dang et al. [25,26] show that by increasing the weight percentage of Halloysite, the strength of the epoxy-Halloysite nanocomposite and the vinyl ester-Halloysite nanocomposite will be significantly increased.

In this study, in-situ polymerization was used to prepare HNT polyurethane composites. Also, the effect of the mixing sequence of monomers for investigating hard segment content and size with the time of adding nanoparticles and its effect on the physical and polyurethane physical properties and phase separation is the subject studied for the first time and produces samples with different properties. The effect of polyurethane chain length in dispersion of nanotubes along the in-situ polymerization process was studied. Distribution and dispersion of nanotube in the kinetic of phase separation is investigated.

## 2. Experimental

### 2.1. Materials

The HNTs were purchased from Sigma-Aldrich and were used without any purification process. PTMG was supplied by Sigma-Aldrich Company and has a purity of 99%. HDI from Aldrich Company has a purity of 99% and has been used without any additional operations. 1, 4_butanediol, one of the four stable butanone isomers, has been used to increase the polyurethane molecular weight. This material was purchased from Aldrich Company. Dimethylacetamide (DMAc) with a purity of 99% was used without any additional operations and was supplied by Sigma-Aldrich.

### 2.2. Preparation of polyurethane nanocomposites

For the synthesis of polyurethane, the oil bath was first introduced to a temperature of 80 ° C so that all of the reactants react at that temperature at the specified times. The reactor was then placed in a three-span oil bath, and for each sample was added 15 ml of dimethylacetamide solution. In each sample, the nanoparticles are sonicated to the reactor for 20 minutes in 5 ml of the solvent and then added to the medium.

Sample one (PU1): Initially, 10 g of PTMG is added to the reactor, then add 2.52 g HDI as a drop into the system and allowed to react for 3 hours so that all hydroxyl groups are grouped with isocyanate It is consumed and urethane pre-polymer. After 3 hours, 0.9 grams of BDO are added to the reactor to complete the reaction for 3 hours.

Sample two (PU2): 10 g PTMG is added to the reactor and then, 135 g HNTs. After mixing in the solvent well by the stirrer, an additional 2.9 g HDI is added to the system as a drop in the system and allowed to react for 3 hours. Add 0.9 g BDO to the reactor dropwise, so that the reaction is complete for 3 hours.

Sample three (PU3): 10 g of PTMG is added to the reactor, then add 2.92 g HDI to the system and allow to react for 3 hours until all hydroxyl groups are consumed by the isocyanate groups And pre-polymeric urethane. At this stage, a quantity of 135.0 g of HNT is added to the reactor, and an hour of reaction is allowed to proceed. In the end, add 0. 9 g to the reactor dropwise, so that the reaction is complete for 3 hours.

**Figure 1.**
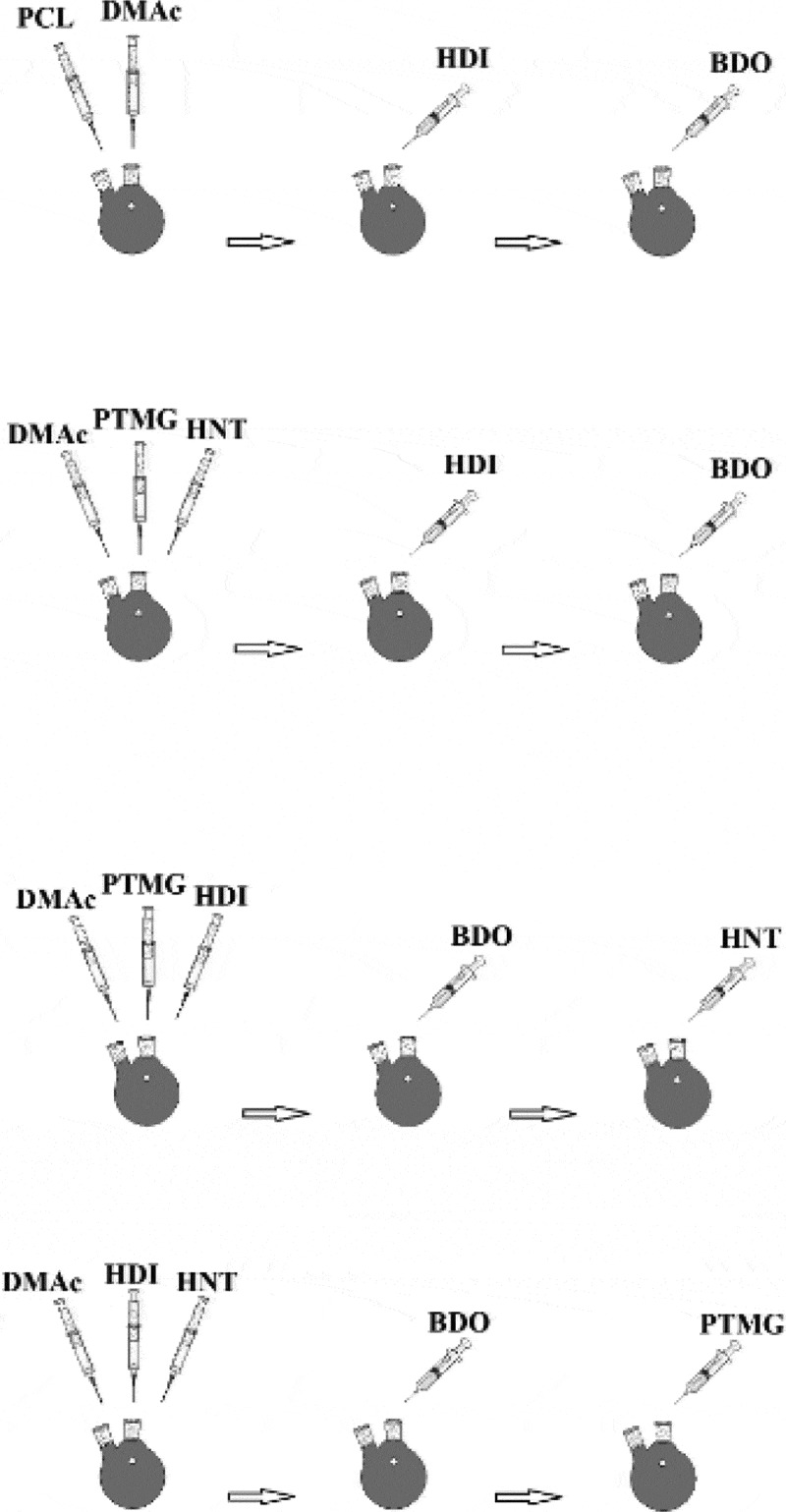
The schematic illustration of the mixing procedure.

Sample four (PU4): An excess amount of (2.92 g) HDI is added to the system. Then,135.0 g of HNTs are added to the reactor and allow an hour of reaction to proceed. 0.9 g of BDO is added to the reactor dropwise in order to complete the reaction for 3 hours. Finally, 10 g PTMG is added to the reactor and allow the reaction to complete for 3 hours. The schematic illustration of the mixing procedure is shown in Figure 1.

### 2.3. Characterization

Attenuated Total Reflection Fourier transform infrared spectroscopy (ATR-FTIR) is an effective method for analyzing chemical bonds and functional groups of Synthesized TPU and nanocomposites. This test was performed using the Thermo Nicolet and the Nexus670 model. The range of wave numbers cm-1 is 4000–600. The type of crystal and angle used is ZnSe, and 45 degrees, respectively. To investigate the effect of nanoparticles on the melting point and crystallinity of nanocomposites Differential scanning calorimetry (DSC) NETZSCH DSC 200 F3 is used with a heating rate of 10 ° C/min under nitrogen atmosphere. The operating temperature range varied from −20 ° C to + 200 ° C. The calibration of the device was carried out using indium and zinc standards. Scanning electron microscopy (SEM) was also used to observe nanoparticles and their distribution in the polymer matrix. For this purpose, the field emission scanning electron microscopy of the Sigma model was used. The physical vapor deposition (PVD) method was used to coat the surface of the samples with gold made by BAL-TEC, a Swiss company. The dynamic mechanical analysis was conducted on the DMA Tritec 2000 device manufactured by TRITON UK. Samples with dimensions of 25 × 10 × 1 mm were prepared for the test. Tanδ and storage modulus at a temperature range 80–40 ° C, heating rate 4 ° C/min and frequency 1 Hz was measured under the nitrogen atmosphere in the tension mode. The cooling operation was performed by liquid nitrogen. To investigate the rheological behavior, a mechanical rheometer (Paar Physica USD 200) was used. Spindle parallel plates with a diameter of 25 mm and a gap between 1 mm were used. To avoid decomposition of the samples, all tests were carried out under nitrogen atmosphere. each specimen were preheated at a temperature of 200 ° C for 10 minutes and then, enter the stress at a rate of 5 for 15 seconds and complex viscosity in terms of the frequency plotted. The frequency range was from 1000 to 0.1 Hz. After this test, the samples are immediately cooled down to 140°C in 3 minutes, and the time sweep was taken. The mechanical properties of different specimens were measured using an electromechanical stretching device (Instron 5566, Elancourt, France). The rectangular specimens were 6 cm long and 1 mm wide manually separated from the specimens. After measuring the thickness with the Thickness Gauge Model B-2, all specimens were installed between two jaws at a distance of 3 cm. The strain-stress curves of these specimens were obtained at a pulling speed of 40 mm/min. This test was performed for at least three samples and the significance of the samples was examined. The value of P < 0.05 was considered significant.

## 3. Result and discussion

### 3.1. Characterization

**Figure 2.**
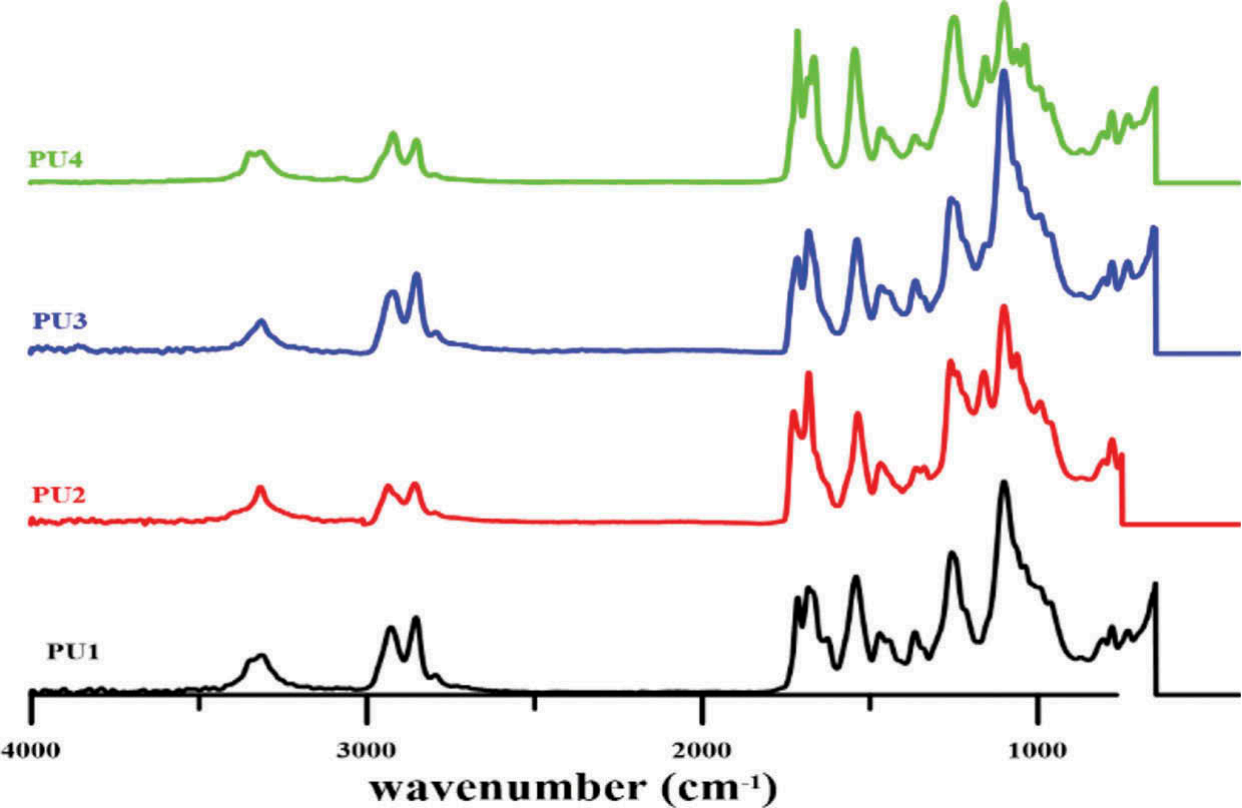
ATR-FTIR result of pure plyurethane and nanocomposites.

The FTIR spectrum is shown in Figure 2. Peaks at 3699 and 3625 cm^−1^ assigned to the Al-OH stretching vibration. The peak at the 910 cm^−1^ is related to the bending of the Al-OH. Peak 1094 cm^−1^ corresponds to the Si-O bond, while the peak 1033 cm^−1^ is related to Si-O-Si cm^−1^ vibrations and 534 is also attributed to the vibration of the Al-O-Si bond in halloysite nanotubes. The peaks observed at 2740 and 2854 cm^−1^ are associated with asymmetric and symmetric stretching vibrations of PTMG, respectively. Other peaks related to the C-O-C bond appeared at 1053 cm^−1^ and CH at around 1500 cm^−1^ and 2900 cm^−1^. The absence of the peak of the isocyanate functional group at around 2270 cm^−1^ implies the consume of all these functional groups during the reaction. The carbonyl peaks which includes hydrogen-bonded carbonyl and non-hydrogen-bonded carbonyl groups appeared at 1685 and 1730 cm^−1^ respectively. Hydrogen-bonded NH groups which interacted with carbonyl group or ether group of PTMG at 3324 cm^−1^ and non-hydrogen-bonded NH groups at 3350 cm^−1^ have appeared.

By measuring the ratio of the area of hydrogen-bonded carbonyl to free carbonyl peak, the hydrogen bonding index (R) in the polyurethane can be obtained from the FTIR graph, and through R/R + 1, the degree of phase separation in the polyurethane nanocomposites can be determined. Via increasing R, the degree of phase separation increases in polyurethane nanocomposites as shown in Table 1. For PU2, the R ratio has reached the maximum value. Therefore, it can be concluded that it exhibits the highest phase separation compared to other samples. The R ratio in PU3 is lower than PU2. Therefore, it can be argued that due to the better distribution of nanoparticles in the PU2 more hydroxyl groups in halloysite nanotubes are available to form hydrogen bondings with carbonyl groups, and the phase separation in this PU2 is greater than that of the three specimens. To further examine this hypothesis, in later sections, images of scanning electron microscopy will be investigated. Interestingly, this ratio in PU1 is higher than PU4 which containing HNTs. Thus, in PU4, hard segments cannot move toward each other to form hard phases, even though to the thermodynamic incompatibility with soft segments. It was expected that in the presence of HNTs with respect to the nucleation mechanism, there would be more phase separation than PU1. However, due to the strong interactions between the polymer chains and the HNTs, the ability to move in PU4 for polymer chains may not exist. The existence of HNTs clusters also hinders the movement of polyurethane chains, and the phase separation PU4, in spite of expectation, reaches minimum value. As seen in PU2 and PU3 due to high phase separation, all NH groups are hydrogen bonded. However, in PU1 and 4, which have fewer phase separation, NH groups are observed which do not have hydrogen bond interactions.

**Table 1.** The values of hydrogen bonding index and the degree of phase separation.

Samples	R	R/R + 1
S1	1.05	0.51
S2	1.55	0.6
S3	1.23	0.55
S4	0.68	0.4

**Figure 3.**
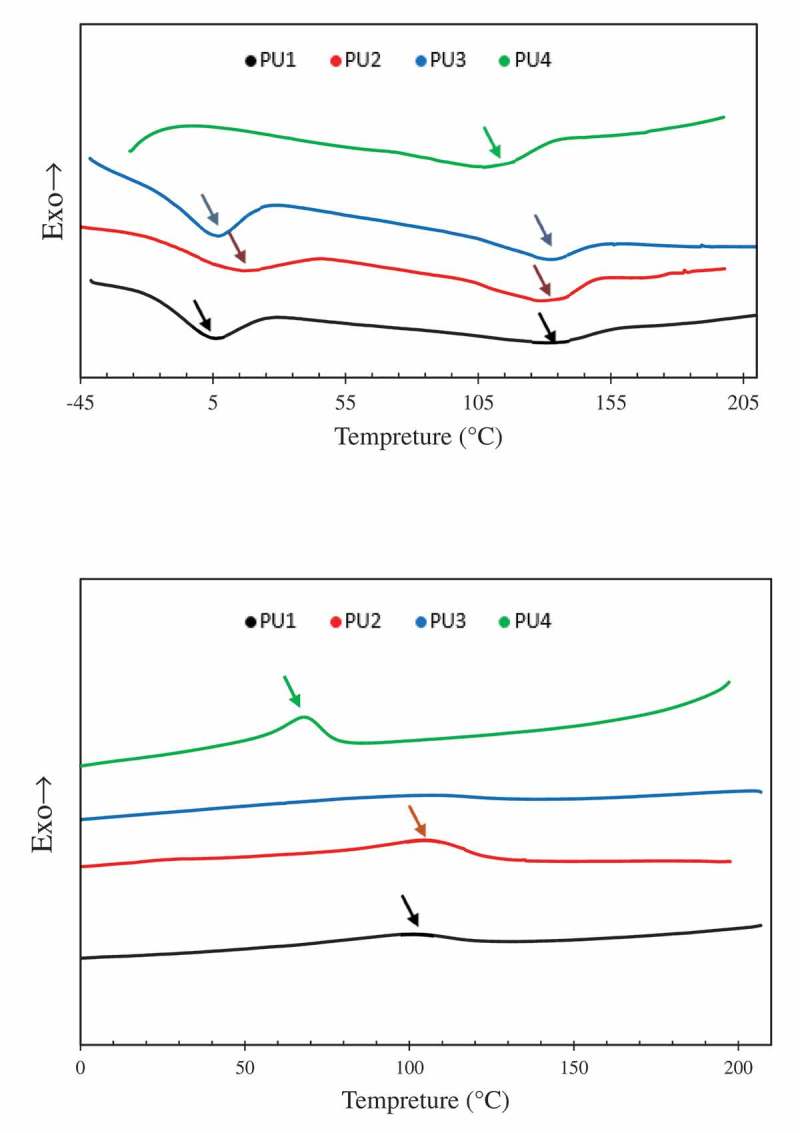
second heating (a) and cooling (b) diagram DSC of samples.

Figure 3(a) shows the second heating and cooling of DSC thermograms. The second heating curves are used because in the first heating process all the defects in the nanocomposites structure are eliminated. As shown in the DSC diagram of the second heating process, the hard and soft phase of PU1, PU2 and PU3 were separately crystallized and showed phase separation.in the DSC thermogram of PU4 there is only one peak between the soft and hard phase crystallization temperatures that show the most phase mixing between the samples. By measuring the amount of ΔH (melting heat) in PU2 and PU3 due to the presence of nanoparticles and their nucleation effect, it is observed that the crystallinity of these samples is higher than that of PU1. Soft phase melting enthalpy of PU2 and PU3 is 11 J/gr and 9.9 J/gr respectively. Which were more than PU1 with 6.84 J/gr as shown in Table 2. in comparison of the PU2 and PU3, due to the better distribution of HNTs in the polyurethane matrix, more nuclei have been formed. Further nucleation increases the crystallinity of the nanocomposites in the hard phase and the ΔH crystallinity of PU2 is more than PU3. In pure polyurethane, the crystallinity temperature of the hard phase iPU132.21°C, which is more than all samples. This means that the thickness of the crystalline layer is higher in pure polyurethane than the other nanocomposites. The hard phase crystallinity temperature was reported for PU2 and PU3 is 128 ° C and 131.36 °C respectively. The presence of nucleating agents such as HNTs in nano-composite systems prevents the growth of crystals. Due to the fact that nanoparticles with nucleation effect increase the amount of crystallinity, but the mechanism of crystallinity in nanocomposites, in addition to nucleation, needs growth. The nucleation phase is accelerated with nanoparticles, but the growth stage in nano-composites is much more difficult due to the presence of nanoparticles, and the crystals formed in nano-composites have a lower thickness. Now, if nanocomposite has a good two-ring pattern, this thickness is less and less and the crystallization temperature reaches its lowest level. For this reason, the PU2 has the lowest melting temperatures among nanocomposites, and PU1 with less crystallinity had the highest melting temperature between PU1, 2 and 3.

**Table 2.** The values from DSC second heating curves.

Samples	T_m_ soft °C	H∆ j.gr^−1^	T_m_ °C	H∆ j.gr^−1^
S1	4.77	15.10	132.21	6.84
S2	15.60	11.74	128.00	11.00
S3	6.60	16.04	131.36	8.90
S4	-	-	106.39	15.39

Figure 3(b) shows the DSC of the samples during cooling. In comparison, PU2,3 TC increased to pure polyurethane and reached fom 99 °C to 100 °C and 105 °C. The PU2 have a higher nucleation effect due to the best dispersion of nanoparticles, and the crystalline temperature reaches a temperature of 105 °C, which is a 6 °C higher than pure polyurethane. In the PU4, the crystalline temperature was much less than the pure polyurethane crystallization temperature because the hard and soft segments were bundled together. However, in this sample Crystallinity is higher than other samples due to the effect of nucleation and chemical interactions between nanoparticles and polyurethane chains. As shown in Table 3, ΔH has reached 10.4 J/gr.

**Table 3.** The values from DSC cooling curves.

Samples	T_c_ °C	H∆ j.gr^−1^
S1	99.25	6.79
S2	105.29	6.54
S3	100.30	7.30
S4	67.70	10.40

**Figure 4.**
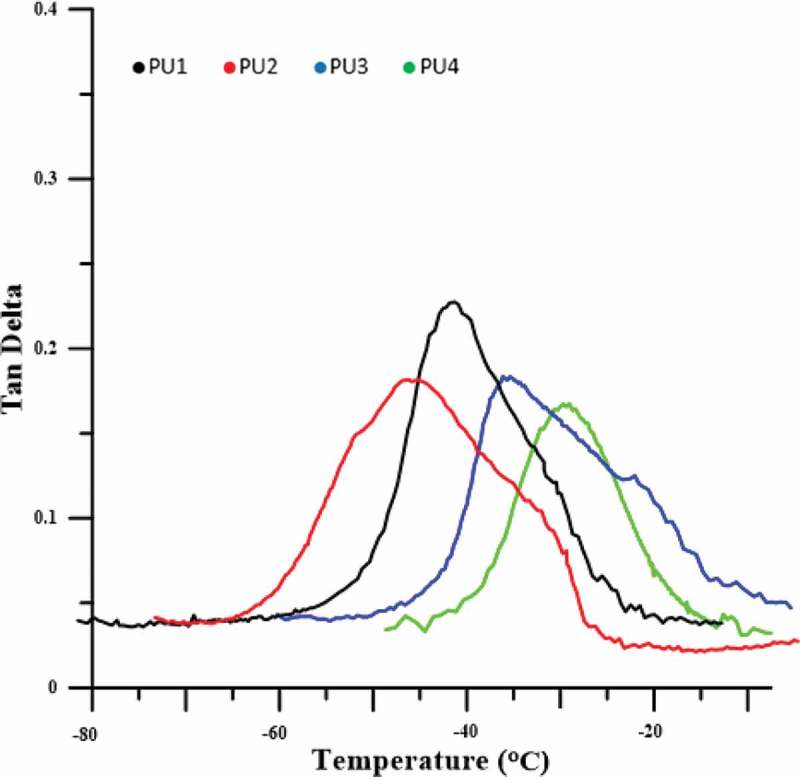
DMTA curves for pure polyurethane and nanocomposites.

To measure the glass transition temperature and the damping factor at glass transition temperature, the DMTA was used (Figure 4 and Table 4). The glass transition temperature peak for pure polyurethane appears at −41.9 ° C. The presence of nanoparticles in pure polyurethane, due to the movement of polymer chains makes harder and creates movement constraints, makes the damping factor of polymer nanocomposites less than pure polyurethane, which In all PU2,3and 4 was significant and shown in Figures 3–4. The damping factor for PU4, which made the interaction between the polyurethane chains and the HNT nanoparticles and highest crystallinity, reaches the lowest value. The glass transition temperatures of PU2 is −46, which is lower than the pure polyurethane. It was expected that in the presence of HNTs, due to the constraints of polymer chains, the glass transition temperature shifts to higher temperatures, but this has not happened.

As shown in the FTIR test, PU2, which has better nanoparticle dispersion and a higher fuzzy separation, the movement of the soft segments is easier and the glass transition temperature has shifted to lower temperatures. But in the PU3 some HNTs cluster in the polyurethane matrix, the parts of the system are frozen. HNTs accumulation, due to the greater size in comparison with hard phase also affects soft segments and the frozen chains in nanocomposites makes the glass transition temperature of this sample shifted to higher temperatures relative to the polyurethane glass transition temperature, which is about 6 °C above the glass transition temperature. The glass transition temperature of PU4 has increased, as compared to PU1 about 10 ° C, due to a large amount of crystallizations that occurred in the nanocomposite and limited the movement of the chains, as shown in the DSC curves. The chemical interactions between polyurethane chains with HNTs has led to a significant increase in the glass transition temperature of soft polyurethane segments. Another reason for this increased glass transition temperature can be attributed to the accumulation of HNTs, which is also larger than the other samples.

### 3.2. Morphological studies

**Figure 5.**
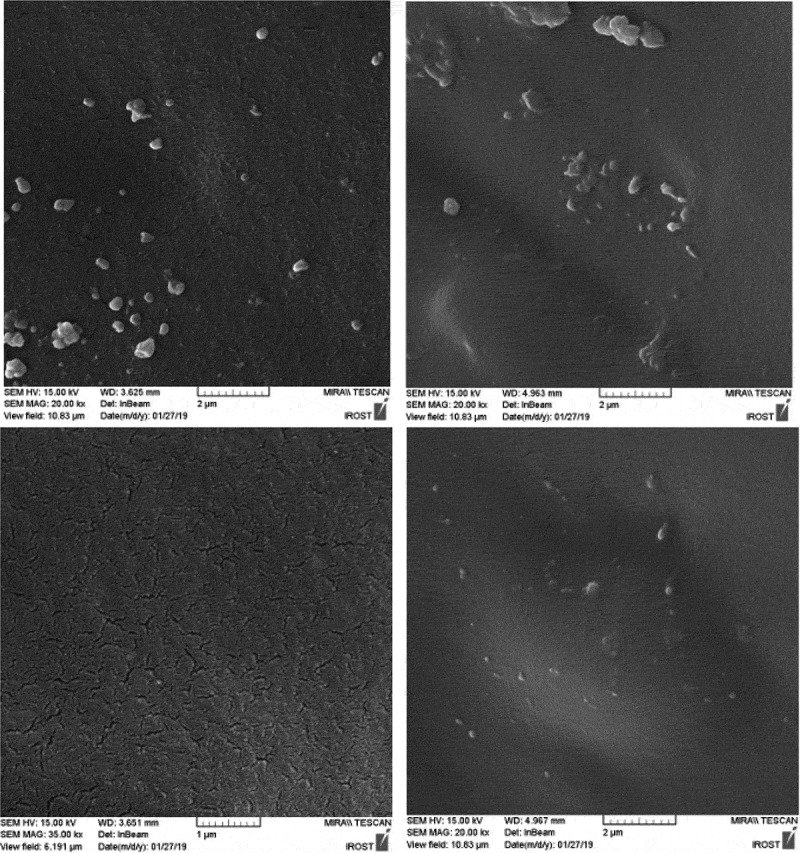
FESEM image of samples in 2 micron scale.

**Figure 6.**
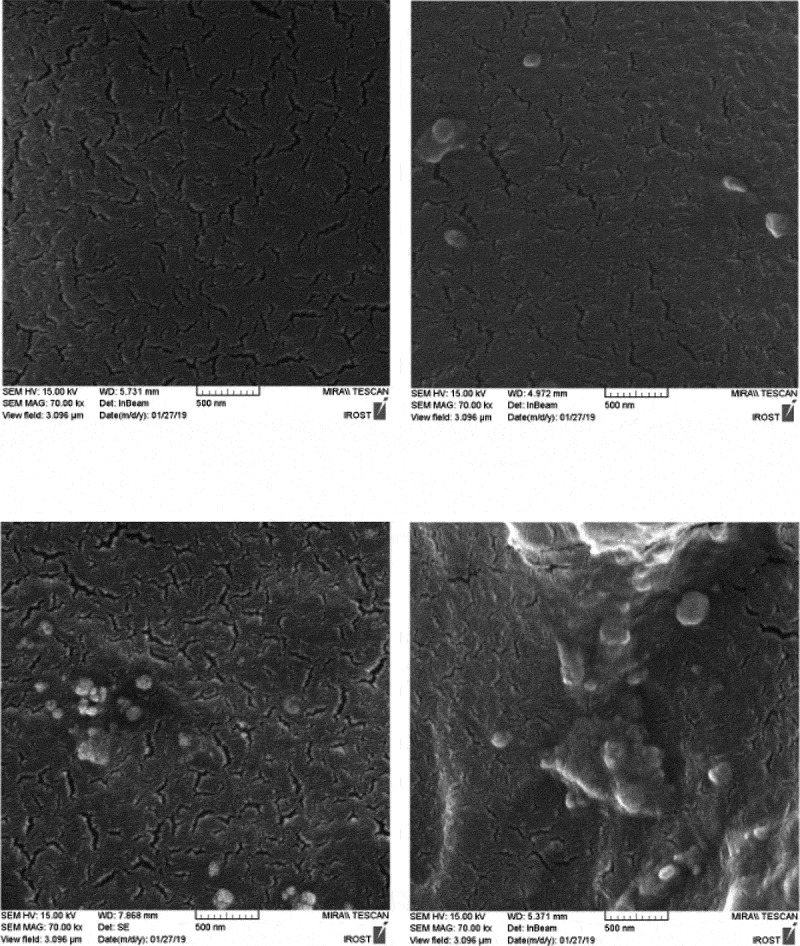
FESEM image of samples in 500 nm scale.

For a closer examination of the morphology and dispersion of nanoparticles, the fracture surface of the specimens in liquid nitrogen was studied by scanning electron microscopy (Figures 5 and 6). These images are shown in Figures 5 and 6 at 2 microns and 500 nm scale. PU2 shows the best dispersion of HNTs. however PU3, some area indicate slight accumulation of HNT nanoparticles and for PU4, because the functional groups on the surface of the nanoparticles have changed and converted to isocyanate, and the surface energy is more intense and tends to be larger, with larger aggregations than the second one, which contains functional groups on the nanoparticles of hydroxyl groups. This interpretation is well illustrated in figures that are shown on a scale of 500 nanometers. Also, the white points that belong to the nanoparticles are bright in the PU3, which means that the interaction between the HNT and the polyurethane chains in this sample is weak and the nanotubes are easily pulled out of the polymer matrix. But in the PU2, due to the stronger interaction between the HNT and the polyurethane matrix, the white points are dark, meaning that polymer chains still exist on nanoparticles and have created more interactions such as hydrogen bonding with nanoparticles.

**Figure 7.**
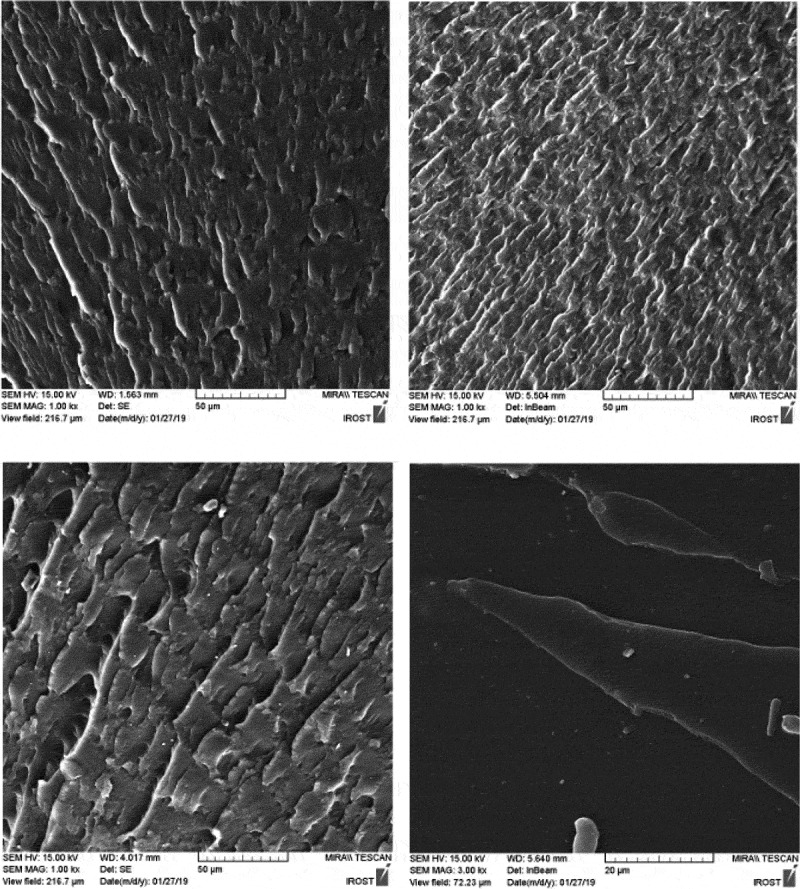
FESEM image of surface fracture after tensile test in 20 micron scale.

The fracture surface of nanocomposites is compared with the pure polyurethane in SEM images (Figure 7). While pure polyurethane showed a very smooth surface, the surface of the nanocomposite was rough to indicate that the accumulation of HNT clusters produced a mechanism of energy dissipation during fracture. Particularly the presence of hard nanotubes changes the fracture pathway and leads to a defect in the polyurethane. Figure 7 shows the fracture surface of the samples from which the tensile test was taken at a scale of 50 μm. As can be seen, the holes in PU1,2 and 3 designate the stiffness of the specimens.as the wrinkles of the sample surface are greater, more toughness is deduced. Therefore, the PU2 is tougher than other specimens. In these images, there are some aggregates exist in PU3,4. However, in PU4 due to its high crystallinity and the creation of chemical interactions, its fracture surface is brittle.

### 3.2. Mechanical investigations

**Figure 8.**
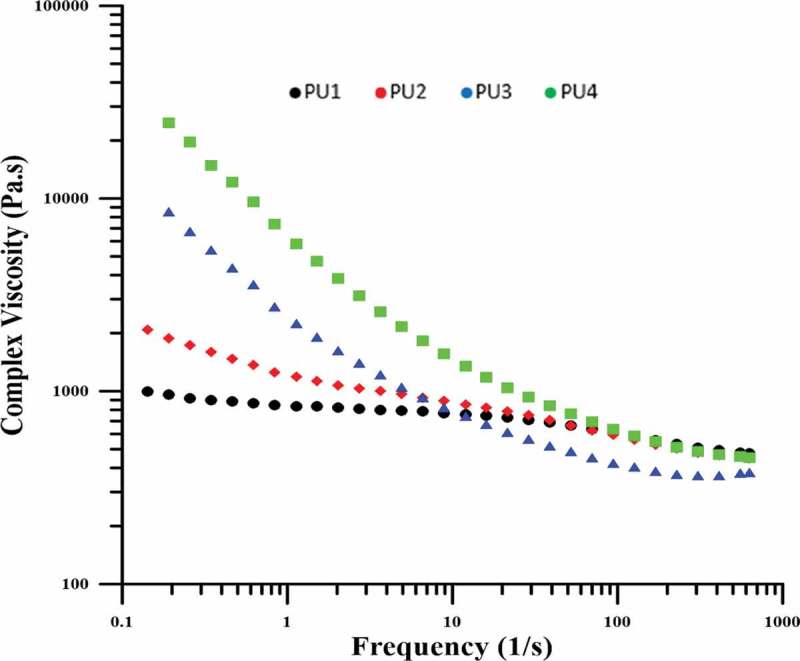
Complex viscosity versus frequency for samples.

As shown in Figure 8, the viscosity of the specimens has been increased by reducing the frequency for all samples. Due to the fact that at low frequencies sufficient time is given to the samples to react or respond to the shear stress, so this response from the polymer chains including the response of high chains and increasing viscosity. But the intensity of this increase was higher for nanocomposites. In nanocomposites, because polymer chains with nanoparticles have physical and chemical interactions, and have a higher limitation to pure polyurethane chains, they have higher viscosity at low frequencies. Among the nanocomposites, PU4, in which some of the polyurethane chains chemically bonded to nanoparticles, exhibit the highest viscosity. However, PU2 has better distribution than PU3 so lower viscosity. The reason for this phenomena is that the nanoparticles in the PU2 are in the polyurethane hard phase and the soft phase can easily move and have a lower viscosity, but in PU3, the presence of HNT clusters causes the regions has a solid-like behavior, and these clusters whose dimensions are larger than the size of the hard segments, also interact with the soft segments which lead to higher viscosity.

**Figure 9.**
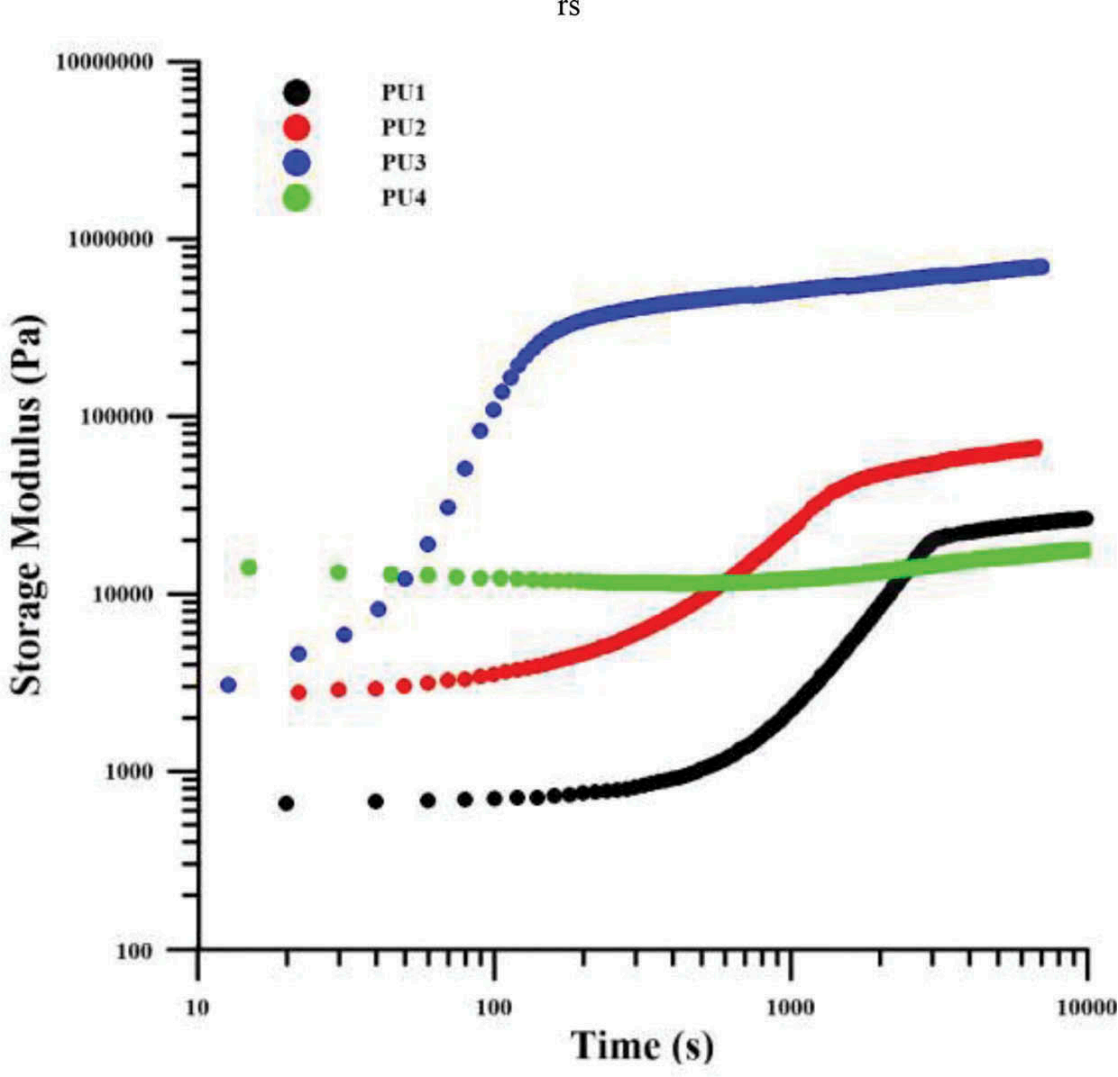
Storage modulus against time for samples.

**Figure 10.**
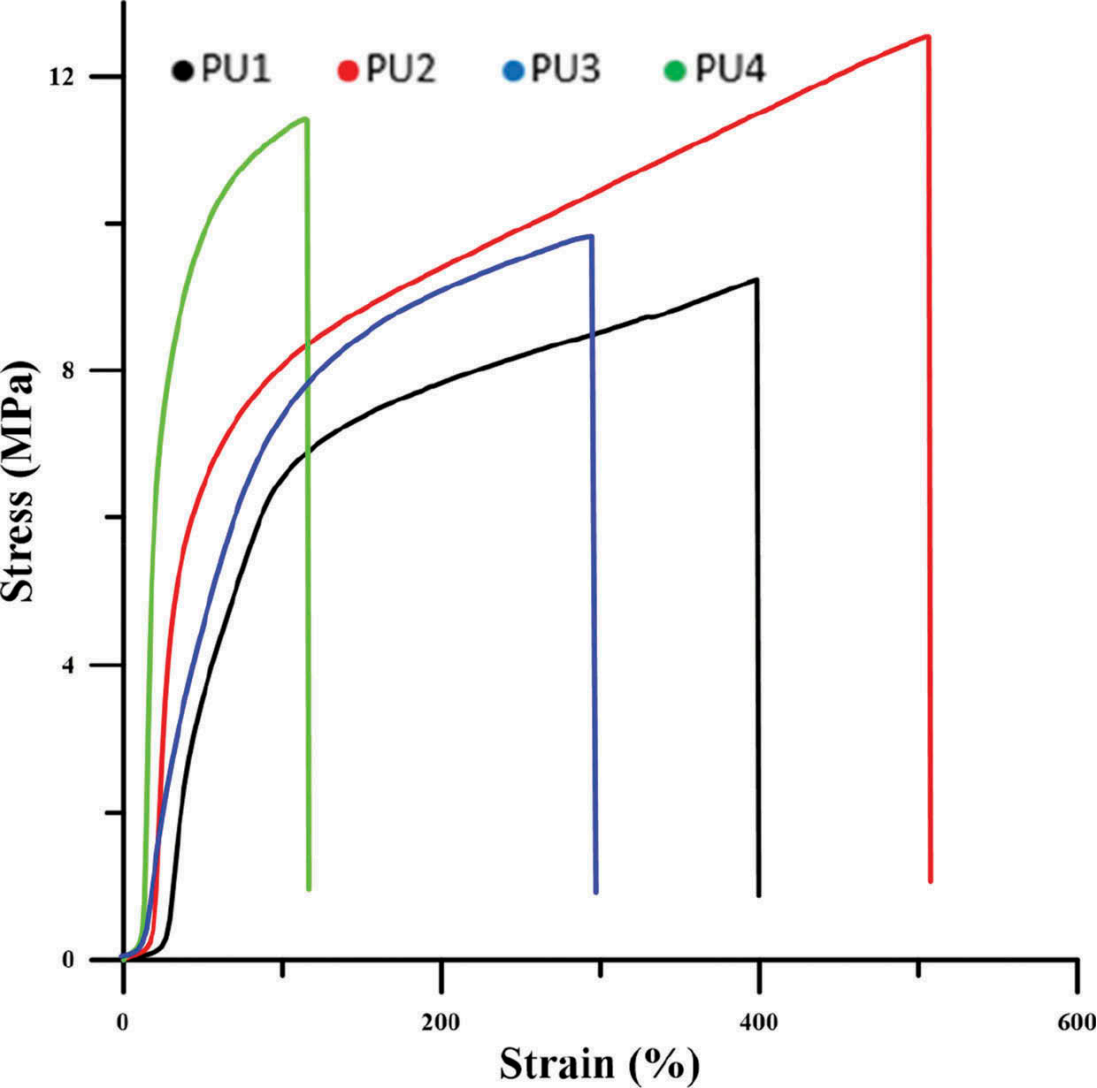
Stress-strain curves.

Elastic modulus variations with increasing time for all specimens are shown in Figure 9 and Table 5. This process is similar to the crystallization process in polymers and consists of two stages of nucleation and growth. According to the diagrams in Figures 3–10, the initial modulus for PU4 is the highest since this sample has the highest hardness among the samples and also, as expected, the pure PU1 has a lower modulus than the nanocomposites at the initial time. Over time, PU4 showed no phase separation and no modulation increase. This behavior confirms the hypothesis of an interaction between polymer chains and nanoparticles in this sample, meaning that polymer chains, although have the tendency to increase phase separation due to thermodynamic incompatibility and have had enough time to do it, Hard segments have not been able to separate from soft segments and the system modulus remains constant. In PU1, the modulus has increased after the phase separation, but the start of phase separation has occurred in subsequent to PU2 and 3, which means that the nucleation in the pure polyurethane occurred later. As expected, HNTs in nanocomposites have caused nucleation in less time. In the PU2, it happened later because nanoparticles were well distributed in the matrix and the polymer chains are not frozen, but in PU3 around the clusters of the HNTs the movement of the chains was completely restricted and the nucleation is more efficient. however, the accumulation of nanoparticles creates enough space for other polymer chains to grow alongside the nuclei, and the growth of the chains is faster than that in which nanoparticles are distributed in the system so the growth rate is higher in PU2 than PU3.A quantitative kinetic study of phase separation has been used from the avrami equations (Equation 1 and 2) and its result is reported in Table 5. In these equations $G{\;}_{0}^{\prime }$ and $G{\;}_{\infty }^{\prime }$, respectively, are the modulus before and after the phase separation. n and k are also the constants of the equation.

(1) $$G^{\prime }\left(t\right)=G{\;}_{0}^{\prime }+G{\;}_{\infty }^{\prime }\left(1-\exp(-kt^{n}\right){)}^{2}$$

(2) $$t_{\frac{1}{2}}=(\frac{\ln2}{k}{)}^{\frac{1}{n}}$$

**Table 4.** Glass transition temperature of pure polyurethane and nanocomposites from DMTA curves.

Samples	T_g_ °C
S1	−41.90
S2	−46.01
S3	−35.20
S4	−29.25

**Table 5.** Initial and final modulus and the rate of modulus increase of samples.

Samples	G’_0_ Pa	G’_∞_ Pa	IG = G’_∞_ – G’_0_/G’_0_	t_0.5_ s
S1	610	26,607	42.61	1333
S2	2511	70,253	26.97	843
S3	3368	852,772	252.19	101
S4	12,808	19,861	0.55	-

Figure 10 shows that HNTs play a significant role in load transfer. at the same strain, the nanocomposites stress is higher than pure polyurethane, indicating that the composite modulus is superior to the pure polyurethane. The stress at the breakpoint is also higher in nanocomposites than pure polyurethane. In PU4, TPU chains that are chemically bonded to nanoparticles so the movement of chains is limited, and the strain at break decreases. In PU2, the greatest amount of energy is spent on the arrangement of nanoparticles and the locking of hydrogen bonds, resulting in an increase in the strain at break t the other nanocomposites. As shown in Figure 10, PU3,4 due to the accumulation of nanoparticles and the creation of weaknesses, the strain at break decreases due to the pure polyurethane. PU3,4 modulus has also increased relative to the pure polyurethane, but due to poor dispersion of the nanoparticles, it has less modulus than the PU2. Tables 6 and 7 are related to tensile strength, elongation to failure, and modulus measured from stress-strain diagrams.

**Table 6.** Tensile strength and Elongation @ break from stress-strain curves.

Samples	Elongation @ break %	Tensile strength MPa
S1	398.8	9.23
S2	507.6	12.52
S3	300.8	9.77
S4	115.9	11.4

**Table 7.** Modulus at 50,100 and 200% starin for samples.

Samples	50 % Modulus MPa	100 % Modulus MPa	200 % Modulus MPa
S1	7.30	6.50	3.90
S2	12.90	8.06	4.70
S3	8.90	7.40	4.60
S4	19.66	11.20	-

## 4. Conclusion

A successful synthesis of polyurethane was deduced from the formation of carbonyl and NH groups and the lack of isocyanate peak from the FTIR diagrams. Also, the sample that was best dispersed has the most phase separation, but the samples also had HNT clusters, and the chemistry interactions with nanoparticles have the least phase separation. In all of the DSC diagrams, the peaks of the crystallinity of the soft and hard phases emerged separately, but in the PU4, due to the phase separation, a peak between the melting temperatures of the soft and hard segments appeared. Also, PU2, which had better distribution, had the highest crystallinity than PU1 and 3, but due to incomplete crystals, it displayed the lowest crystalline temperature. SEM images showed that the nanoparticle dispersion in PU2 was better than PU3 and 4. Therefore, the fracture surface of the PU1, 2, and 3, were rough and for the PU4, it was smooth DMTA charts showed that the loss factor in samples containing nanoparticles decreased compared to pure polyurethane. Also, the glass transition temperature of nanocomposites is higher than pure polyurethane except in a sample that has a well-dispersed nanoparticle that has the lowest glass transition temperature. Rheological tests also showed that the presence of nanoparticles accelerates phase separation. PU3 had the largest and fastest phase separation. However, in S 4, fuzzy separation was not observed due to the confinement of polymer chains. The results of stress-strain tests also show that the PU2, while increasing the modulus and the tensile strength of the net sample, had the highest elongation at break, indicating that HNTs act as a reinforcing agent in the polyurethane matrix. PU4 showed the highest modulus and tensile strength. This was due to the crystallinity and rigidity of the chains of this sample.
